# Editorial board

**DOI:** 10.1080/0886022X.2023.2310882

**Published:** 2024-03-27

**Editor-in-Chief**

**Tibor Fülöp, MD, PhD** - Medical University of South Carolina, Charleston, South Carolina, USA

**Deputy Editor**

**Mihály Tapolyai, MD, PhD** - St. Margit Hospital, Budapest, Hungary

**Associate Editors**

***Basic Sciences***

**Shao-bin Duan, MD, PhD** - Central South University, Changsha, China

**Monika Gööz, MD, PhD** - Medical University of South Carolina, Charleston, South Carolina, USA

**Guisen Li**; **MD, PhD** - School of Medicine, University of Electronic Science and Technology of China; Chengdu, China

***Clinical Sciences***

**Fan He, MD, PhD** – Tongji Medical College of Huazhong University of Science and Technology, China

**Sohail Abdul Salim, MD** – University of Mississippi Medical Center, Jackson, Mississippi, USA

**Michael E. Ullian, MD** – Medical University of South Carolina, Charleston, South Carolina, USA

**Guangtao Xu**, **MD** – Jiaxing University, Jiaxing, Zhejiang, China

***Critical Care and Perioperative Sciences***

**Csaba Kopitko, MD, PhD** - Uzsoki Utcai Kórház – Semmelweis University, Budapest, Hungary

***Transplantation Sciences***

**Karim M. Soliman, MD, PhD** - Medical University of South Carolina, Charleston, South Carolina, USA

***Clinical Epidemiology, Meta-Analyses and systemic Reviews***

**Wisit Cheungpasitporn, MD -** Mayo Clinic, Rochester, Minnesota, USA

**Wisit Kaewput, MD** - Phramongkutklao College of Medicine, Bangkok, Thailand

***Biostatistics* and Statistical Consultant Advisor:**

**Zsolt Lengvárszky, PhD** - Louisiana State University Shreveport; Shreveport, LA, USA

**Editorial Board Members**

**Ahmed I. Adelkader, MD, PhD -** Medical University of South Carolina, Charleston, South Carolina, USA

**Anand Achanti, MD** - Medical University of South Carolina, Charleston, South Carolina, USA

**Adam Ryan, PhD** – Medical College of Wisconsin, WI, USA

**Gaetano Alfano, MD** – University Hospital of Modena, Modena, Italy

**Aysen Akalin, MD** - Eskisehir Osmangazi University, Eskisehir, Turkey

**Hatem Ali, MBBCh, MSc** - University Hospitals of Coventry and Warwickshire, Coventry, UK

**Andrea Angioi, MD** - Azienda Ospedaliera G. Brotzu, Cagliari, Italy

**Petar Avramoski, MD, PhD** - St. Clement of Ohrid University, Bitola; Clinical Hospital – Bitola, North Macedonia

**Sebastjan Bevc, MD, PhD** - University Medical Center Maribor, Maribor, Slovenia

**Ana de Lurdes Agostinho Cabrita, MD** - University Hospital Center of Algarve, Faro, Portugal

**Jorge Castaneda, MD** - Banner University Medical Center - Phoenix, Phoenix, Arizona, USA

**Alejandro R. Chade, PhD** - University of University of Missouri-Columbia, Columbia, MO, USA

**Jin-Bor Chen, MD** - Kaohsiung Chang Gung Memorial Hospital, Chang Gung University College of Medicine, Kaohsiung, Taiwan

**Orsolya Cseprekál, MD, PhD** - Semmelweis University, Budapest, Hungary

**Sihem Darouich, MD, MSc** - University of Tunis El Manar, Tunis, Tunisia

**Mehul P. Dixit, MD** - University of Mississippi Medical Center, Jackson, Mississippi, USA

**Neville R. Dossabhoy, MD** - University of Mississippi Medical Center, Jackson, Mississippi, USA

**Emre Eşkazan, MD** - Istanbul University-Cerrahpaşa, Istanbul, Turkey

**Youssef Farag, MD, PhD, MPH**- Harvard Medical School, Boston, Massachusetts, USA

**Liliana Gadola, MD** – Universidad de la Republica Facultad de Medicina, Montevideo, Uruguay

**Abduzhappar Gaipov, MD, PhD** - Nazarbayev University School of Medicine, Nur-Sultan City, Kazakhstan

**Xuxia Gao, MD, PhD** - Capital Medical University Affiliated Beijing Anzhen Hospital, Beijing, China

**Mohammed Abdel Gawad, MD, ESENeph** - Newgiza University, Giza, Egypt

**S. Mehrdad Hamrahian, MD** - Thomas Jefferson University, Philadelphia, Pennsylvania, USA

**Nicolas Hanset, MD** - University Hospital Saint-Luc, Brussels, Belgium

**Fan He, MD, PhD** - Tongji Medical College, Huazhong University of Science and Technology, Wuhan City, Hubei Province, P.R.China

**Quan Hong, PhD** - Chinese PLA General Hospital, Beijing, P.R.China

**Bo Hu, MD** - Jiaxing Hospital of Traditional Chinese Medicine, Jiaxing University, Zhejiang, P.R. China

**Jun Jiang, MD** - The First Affiliated Hospital of USTC, University of Science and Technology of China, Hefei, China

**Swetha Rani Kanduri, MD** - Ochsner Medical Center, New Orleans, LA, USA

**Mohamed Khalaf, MD** - East Carolina University, Greenville, North Carolina, USA

**Christian A. Koch, MD, PhD, Medhabil.** - University of Florida, Jacksonville, FL, USA

**Shan Mou, MD, PhD** - Shanghai Jiao Tong University, Shanghai, China

**Miklos Z. Molnar, MD, PhD** - University of Utah, Salt Lake City, UT, USA

**Jolanta Malyszko, MD, PhD** - Medical University of Warsaw, Warsaw, Poland

**Lovelesh Nigam, MD** - Dr. H.L. Trivedi Institute of Transplantation Sciences, Asarwa, Ahmedabad, India

**Surapon Nochaiwong, PharmD** - Chiang Mai University, Chiang Mai, Thailand

**Amy Perry -** Ralph H. Johnson VAM Medical Center, Charleston, SC, USA

**Ákos G. Pethő, MD, PhD** – Semmelweis University, Budapest, Hungary

**László Rosiváll, MD, PhD, DSc Med, Medhabil** - Semmelweis University, Budapest, Hungary

**Ken Sakai, MD, PhD** - Toho University Faculty Medicine, Tokyo, Japan

**László Szabó, MD** - University Hospital of Wales, Cardiff, UK

**J. Pedro Teixeira, MD** - University of New Mexico, Albuquerque, NM, USA

**Bijin Thajudeen, MD**- Banner University of Arizona, Tucson, AZ, USA

**Charat Thongprayoon, MD**- Mayo Clinic, Mankato, MN, USA

**Ningning Wang, MD, PhD** - The First Affiliated Hospital of Nanjing Medical University, Nanjing, China

**Yong-Gui Wu, MD** - Anhui Medical University, Hefei, China

**Jianyun Yan, PhD** - Zhujiang Hospital of Southern Medical University, Guangzhou, China

**Fan Yi, PhD** - Shandong University, Jinan, Shandong, China

**Hongliang Zhang, MD, PhD** - National Natural Science Foundation of China, Beijing, China

**Zhongheng Zhang, MD** - Sir Run Run Shaw Hospital, Zhejiang University School of Medicine, Hangzhou, P.R. China

**Previous Editor:**

**William F. Finn, MD** - University of North Carolina, USA

